# The increasing presence and relevance of the shoulder in European Society for Sports Traumatology, Knee Surgery and Arthroscopy

**DOI:** 10.1002/jeo2.70299

**Published:** 2025-07-13

**Authors:** Miguel Angel Ruiz Iban, Berte Böe, Emmanouil Brilakis

**Affiliations:** ^1^ Orthopaedic Surgery and Trauma Service Hospital Universitario Ramón y Cajal Madrid Spain; ^2^ Departamento de Cirugía, Ciencias Sanitarias Y Medicosociales Universidad de Alcalá de Henares Alcalá de henares Madrid Spain; ^3^ Division of Orthopaedic Surgery Oslo University Hospital Oslo Norway; ^4^ 2nd Vicepresident of ESSKA Luxembourg; ^5^ BLAKIS ORTHOPAEDICS Athens Greece; ^6^ 3rd Orthopaedic Department of Hygeia Hospital (Approved ESSKA Teaching Centre) Athens Greece; ^7^ Vice‐chair of ESA, a Section of ESSKA Luxembourg

## Abstract

**Level of Evidence:** Level V.

**Abbreviations**
ESA: European Shoulder Associates
ESSKA: European Society for Sports Traumatology, Knee Surgery and Arthroscopy
JEO: Journal of Experimental Orthopaedics

When a young orthopaedic surgery resident is introduced to the bewildering ecosystem of scientific societies in our speciality, she (or he) is soon aware of European Society for Sports Traumatology, Knee Surgery and Arthroscopy (ESSKA), a cornerstone among sports‐related societies in Europe and around the World: ‘ESSKA is involved in all aspects of sports orthopaedics, from arthroscopy of any joint to sport‐specific medicine, including, of course, a lot of knee surgery, including arthroplasty’. This resident probably thinks the shoulder is included in the mix: ‘There are not one but two “S” in the title, so one must be for shoulder….’. But a senior fellow soon will prove him (or her) wrong: ‘Shoulder surgeons are a quite special lot; although they deal with a lot of sport‐related injuries and, indeed, they perform a lot of arthroscopic procedures, they also tend to support only a few strong, independent, national, European and worldwide shoulder societies’. This same budding resident will eventually later check for herself (or himself) and find that many top shoulder surgeons are ESSKA members, ESSKA supports a lot of shoulder‐based activities, the ESSKA congress has a dozen ‘shoulder focused’ sessions, and within the society, there is a specific section focused on the shoulder: European Shoulder Associates (ESA).

The birthing of ESSKA was humble but ambitious in the divided Berlin of 1982. The founding of the society was complex and fraught with uncertainties. Still, the leadership of Dr. Ejnar Erickson and the ingenuity of the newly founded board members made the society instantly successful with surgeons, attracting 800 participants in the first congress in Berlin in 1984 [3]. The minutes of the first Board meeting, signed on 1 September 1982, do not mention the shoulder at all, as the focus was the knee and arthroscopic techniques [4], and shoulder arthroscopy was only nascent at that time [5]. Things changed quickly, and, already in the 6th ESSKA Congress in Berlin 1994, there were two sessions dedicated to the shoulder, one focused on arthroscopic surgery, and another focused on shoulder instability, at that time a mainly nonarthroscopy issue [2].

If one should select a pivotal moment in the history of the shoulder in ESSKA, it would most certainly be the founding of the ESA section in 2010, with the initial leadership of Pascal Gleyze [3]. The development of this section, with a focus on all things shoulder and elbow and a friendly relationship with the European Society for Surgery of the Shoulder and the Elbow, helped to define the key role that shoulder surgery had in ESSKA. ESA has been highly active inside ESSKA: it supports the development of the Congress agenda, participates in the Speciality Days, and promotes biennial closed meetings (newly renamed as Focus meetings). More recently, it has embraced the push for high‐quality, evidence‐based, knowledge, crystallised in ESSKA Consensuses, with an already published shoulder instability consensus [6] and another focused on partial posterosuperior cuff tears in development.

ESSKA's commitment to shoulder surgery is not limited to research and meetings. Recognising the importance of structured education, ESSKA offers dedicated courses for shoulder surgeons and has established certification pathways in key areas such as shoulder instability and rotator cuff surgery. These programs provide young surgeons with a unique opportunity to benchmark their skills against the highest European standards, reinforcing ESSKA's role as a leader in orthopaedic education.

This journal is not impervious to the leading role the shoulder is playing in ESSKA. *The Journal of Experimental Orthopaedics* (JEO), from the very beginning in 2014, embraced shoulder‐related papers [7]. This trend has been followed: in 2024, 10% of all research published in JEO focused on the Shoulder or Elbow, a percentage maintained from the journal's beginning. Since 2019, it has started publishing papers on shoulder arthroplasty [1], a topic traditionally less prioritised by its sister journal, KSSTA.

The shoulder has come to stay in ESSKA and JEO, and we all embrace this subspeciality with joy and pride, recognising that a strong shoulder and elbow community enriches the entire ESSKA family. If you are a resident or young orthopaedic surgeon with a budding interest in shoulder surgery, do not hesitate to join us. ESSKA is here to teach you, certify you, publish your best research, and help you become a more skilled and successful surgeon, always with the ultimate goal: to be a better surgeon, to help your patients by offering them the highest quality care, which should always be the focus of our practice.

## CONFLICT OF INTEREST STATEMENT

The authors declare no conflicts of interest.

## ETHICS STATEMENT

The authors have nothing to report.
